# A Novel Predictive Model in Recognizing Severe COVID-19 and Multiorgan Injuries: Platelet-to-CRP Ratio

**DOI:** 10.1155/2022/6549399

**Published:** 2022-05-10

**Authors:** Wei Chen, Kenneth I. Zheng, Saiduo Liu, Chongyong Xu, Chao Xing, Zengpei Qiao

**Affiliations:** ^**1**^ Department of Radiology, The Second Affiliated Hospital of Wenzhou Medical University, Yuying Chilldren's Hospital, Wenzhou, China; ^2^MAFLD Research Center, Department of Hepatology, The First Affiliated Hospital of Wenzhou Medical University, Wenzhou, China; ^3^Department of Infectious Disease, The Sixth People's Hospital of Wenzhou, Wenzhou, China; ^**4**^ Department of Laboratory Medicine, The Second Affiliated Hospital of Wenzhou Medical University, Yuying Children's Hospital, Wenzhou, China

## Abstract

**Aims:**

In view of the emerging virus variations and pandemic worldwide, it is urgent to explore effective models predicting disease severity.

**Methods:**

We aimed to investigate whether platelet-to-CRP ratio (PC ratio) could predict the severity of COVID-19 and multi-organ injuries. Patients who complained of pulmonary or gastrointestinal symptoms were enrolled after confirmation of SARS-CoV-2 infection via qRT-PCR. Those who complained of gastrointestinal symptoms were defined as having initial gastrointestinal involvement. Chest computed tomography (CT) was then performed to classify the patients into mild, moderate, and severe pneumonia groups according to the interim management guideline. qRT-PCR was also performed on stool to discern those discharging virus through the gastrointestinal tract. Logistic regression models were applied to analyze the association between PC ratio and severity of pneumonia, risk of initial gastrointestinal involvement, and multi-organ injuries.

**Results:**

When compared to the bottom tertile of PC ratio, the adjusted odds ratio was −0.51, *p* < 0.001 and −0.53, *p* < 0.001 in moderate and severe pneumonia, respectively. Furthermore, the adjusted odds ratio for initial gastrointestinal involvement was 0.18 (82% lower) when compared to the bottom tertile of PC ratio, *p*=0.005. The area under ROC on moderate-to-severe pneumonia and initial gastrointestinal involvement was 0.836 (95% CI: 0.742, 0.930, *p* < 0.001) and 0.721 (95% CI: 0.604, 0.839, *p*=0.002), respectively. The upper tertiles of PC ratio showed lower levels of aspartate aminotransferase (*p*=0.016) and lactic dehydrogenase (*p* < 0.001).

**Conclusions:**

Platelet-to-CRP ratio could act as an effective model in recognizing severe COVID-19 and multi-organ injuries.

## 1. Introduction

The virus responsible for the epidemic worldwide that started in late 2019 (COVID-19) has been identified as novel coronavirus by the World Health Organization (WHO) and termed as SARS-CoV-2, characterized as highly contagious and deadly [1]. Numerous researches have focused on the pandemic; however, the particular mechanisms responsible for the nearly 20% hospitalization rate and over 5.4 million deaths globally attributed to SARS-CoV-2 infection at the time of writing remain insufficiently understood [2, 3]. Therefore, to develop efficient models to predict COVID-19 progression and clinical manifestations remains urgent.

Studies showed that, during inflammation caused by viral or bacterial infections, platelets usually play a critical role in forming barrier forbidding infection dissemination, which modulated the physiological and pathological responses against infection [4, 5]. Several researches have shown that SARS-CoV-2 infection would likely activate platelet, inducing an aggressive hypercoagulable state consisted of deep vein thrombosis and pulmonary embolism [6], though treated with standard anticoagulant therapy. And other findings about the C-reactive protein (CRP) revealed a positive correlation with the severity of COVID-19 [7, 8].

Based on these findings, we hypothesized that platelet-to-CRP ratio (PC ratio) could act as a package solution in predicting COVID-19 progression and multi-organ injuries. And to our knowledge, this is the first study to develop a package solution in discerning severe COVID-19 from mild or moderate ones.

## 2. Material and Method

### 2.1. Study Design and Participants Selection

The current bicentral research protocol conformed to the ethical guidelines of the 1975 Declaration of Helsinki, which was further authorized by the local ethics committee of the Second Affiliated Hospital of Wenzhou Medical University and the People's Sixth Hospital of Wenzhou. Written informed consent has been archived from each participant.

According to the policies against COVID-19 pandemic, the participants with the complaints of pulmonary manifestations (chest pain, cough, dyspnea, for instance), or extrapulmonary symptoms (vomiting, diarrhea, for instance), were swabbed and screened by qRT-PCR assay for SARS-CoV-2 nucleic acid. Based on the CDCP (China) guidance in diagnosis and management of COVID-19, those with positive qRT-PCR signals were enrolled [9]. Those who could not obey the protocol to undergo the computed tomography (CT) detection or blood sample collection were excluded.

The involvement and injuries of extrapulmonary organs were assessed with clinical manifestations, or serological tests, including transaminase, total bilirubin, lactic dehydrogenase, and albumin, while the involvement of gastrointestinal (GI) was defined as positive GI symptoms at admission.

### 2.2. Thorax Computerized Tomography (CT) and Judgement

Thorax CT inspections were carried out in a single inspiratory phase, according to the protocol provided by the CT scanner manufacturer (Optima CT540, GE Healthcare, USA), just as reported previously [7]. Briefly, participants were guided to breath-keeping to prevent the motion-artifacts. Raw data for CT images were obtained by the following protocols: valid tube voltage of 100–120 kVp; valid tube electric current of 110–250 mAs; detector collimation of 0.625 mm; slice thickness and interval of 1 mm and 0.8 mm, respectively. Based on the raw data collected above, the thorax CT images were displayed after reconstructed iteratively.

**Figure 1:**
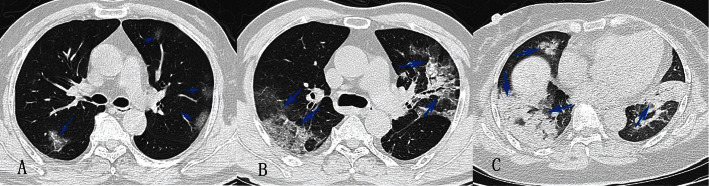
CT grading. (a) Mild pneumonia (peripheric and subpleural damping as ground glass). (b) Moderate pneumonia (thick shadow with multiple lung lobes (≥3) affected; cloudy flocculent or stone like lesions; local lobes consolidation (≥2); lobes fibrosis or sign of air bronchograms). (c) Severe pneumonia (consolidation with a minimum of 80% of the pulmonary or 4 lobes involvement; strip-shaped lesion and significant fibrosis).

Two excellent radiologists, Wei Chen and Chongyong Xu, who have more than ten years of experience in thoracic radiology, performed the image judgement and lesion grading individually. Then, a final score and lesion grading for each participant was achieved by symposium. The pulmonary lesion was recorded as mild (peripheric and subpleural damping as ground glass, Figure 1(a)), moderate (thick shadow with multiple lung lobes (≥3) affected; cloudy flocculent or stone like lesions; local lobes consolidation (≥2); lobes fibrosis or sign of air bronchograms, Figure 1(b)), or severe (consolidation with a minimum of 80% of the pulmonary or 4 lobes involvement; strip-shaped lesion and significant fibrosis, Figure 1(c)).

### 2.3. Blood Sample Collection and Tests

Anonymous venous blood samples were collected in the status of fasting at admission and instantly analyzed in the platform of Mindray BC-5390 (Shenzhen, China), for the purpose of detecting circulating platelet cell, neutrophil, lymphocyte, and the level of CRP and hemoglobin concentration. Serum was separated and stored at −80°C after sampling and then tested in the platform of VITROS 5600 System (VITROS 5600, Johnson, New Jersey, USA), for the purpose of detecting albumin, total protein, lactic dehydrogenase, creatine kinase, alanine aminotransferase, aspartate transaminase, and total bilirubin. All of the tests were carried out in the Second Affiliated Hospital of Wenzhou Medical University.

### 2.4. Assessment of Virus Discharge in Stool

In order to detect viral nucleus in participants' stool, a commercial RNA extract kit (Sol/Insol, BioRad) was applied according to the manufacturer's instructions. Then, total RNA was recovered in 50 *μ*l of elution buffer and instantly used as the template for qRT-PCR system, according to the manufacturer's protocol (Vazyme Biotech Co., Ltd). Briefly, the qRT-PCR amplification system consisted of 2 × One Step SYBR Green Mix (10 *μ*l), One-Step SYBR Green Enzyme Mix (1 *μ*l), 50 × ROX Reference Dye (0.4 *μ*l), 10 *μ*M primer (0.4 *μ*l for each), and RNA template (2 *μ*l). The following settings were applied for targeted sequence amplification: 50°C for 3 min, 95°C for 30 s, followed by a procedure consisted of 40 cycles (95°C for 10 s, 60°C for 30 s for each cycle), and finally completed with a default melting curve procedure in an ABI 7500 platform [10].

### 2.5. Data Statistics

Statistical data analysis was carried out by applying SPSS 25.0 (IBM, New York, U.S.A.). Raw data were finally presented as means ± standard deviations and frequencies for continuous and classified variables, respectively. For continuous variables, one-way analysis of variance (ANOVA) or Student's *t*-test were applied when calculating the differences among the groups of participants, when appropriate. For classified variables, ANOVA, Kruskal–Wallis chi-square test, or Fisher exact tests were applied when calculating the differences among the groups of participants, when appropriate.

In order to explore the associations between PC ratio tertiles and the severity of pneumonia and initial GI involvement, a multivariate linear regression method was applied. A violin boxplot analysis was used to compare the difference in albumin and aspartate transaminase frequentness among the patients of COVID-19 in different tertiles of PC ratio. In order to assess the predicting power of PC ratio on severe pneumonia based on CT performance and the probability of GI involvement, the receiver operation curve (ROC) was applied. In order to explore the best clinical application value of PC ratio, the PC ratio cutoff was calculated according to the largest Youden Index.

## 3. Results

### 3.1. Baseline Characteristics

Finally, 76 COVID-19 patients were enrolled and stratified into PC ratio tertiles (Table 1). As shown in Table 1, the bottom PC ratio tertile had the highest risk of moderate-to-severe pulmonary CT performance (*p* < 0.001) and highest risk of initial GI involvement (*p*=0.017). There were no significant differences in gender constituent and mean age in the three subgroups, indicating a reliable result among the PC ratio tertiles (Table 1). As shown in Table 1, the bottom tertile of PC ratio showed significant higher temperature in the course of COVID-19 (*p*=0.039), higher risk of dyspnea (*p*=0.012), significantly lower lymphocyte count (*p*=0.017), and significantly higher concentration of lactic dehydrogenase (*p* < 0.001) and aspartate transaminase (*p*=0.016). There was no significant difference in the positive rate of qRT-PCR on stool (*p*=0.051). Meanwhile, the bottom tertile showed a significantly higher level of globulin (*p*=0.046) and lower level of albumin (*p*=0.005).

### 3.2. Association between PC Ratio and Pulmonary CT Performance

The precise association between PC ratio and pulmonary CT performance was analyzed using multiple logistic regression model (Table 2). When compared with the 1^st^ tertile, the odds ratio of moderate-to-severe CT performance in the crude model for 2^nd^ and 3^rd^ tertiles decreased by nearly 76% (OR = −0.76 [95% CI: −1.03, −0.49]; *p* < 0.001) and nearly 89% (OR = −0.89 [95% CI: −1.15, −0.62]; *p* < 0.001), respectively. When adjusted for likely confounders, including age, gender, hypertension, total protein, albumin, lymphocyte, lactic dehydrogenase, and lymphocyte, similar association between PC ratio and moderate-to-severe CT performance remained for 2^nd^ tertile (OR = −0.51 [95% CI: −0.78, −0.24]; *p* < 0.001) and 3^rd^ tertile (OR = −0.53 [95% CI: −0.82, −0.24]; *p* < 0.001) when compared to the 1^st^ tertile.

### 3.3. Association between PC Ratio and Initial GI Involvement and Virus Discharge in Stool

The precise association between PC ratio and initial GI involvement was analyzed using multiple logistic regression model (Table 3). When compared with the 1^st^ tertile, the odds ratio of initial GI involvement in the crude model for 2^nd^ and 3^rd^ tertiles decreased by nearly 39% (OR = 0.61 [95% CI: 0.20, 1.89]; *p*=0.391) and nearly 86% (OR = 0.14 [95% CI: 0.03, 0.59]; *p*=0.008), respectively. When adjusted for likely confounders, including age, gender, hypertension, total protein, albumin, lymphocyte, lactic dehydrogenase, and lymphocyte, similar association between PC ratio and initial GI involvement remained for 2^nd^ tertile (OR = 0.94 [95% CI: 0.24, 3.59]; *p*=0.923) and 3^rd^ tertile (OR = 0.18 [95% CI: 0.03, 0.97]; *p*=0.005) when compared to the 1^st^ tertile. Interestingly, no correlation between PC ratio and virus discharge in stool was observed (*p*=0.051) (Table 1).

### 3.4. Predictive Power of PC Ratio on Pulmonary Injuries and Initial GI Involvement

**Figure 2:**
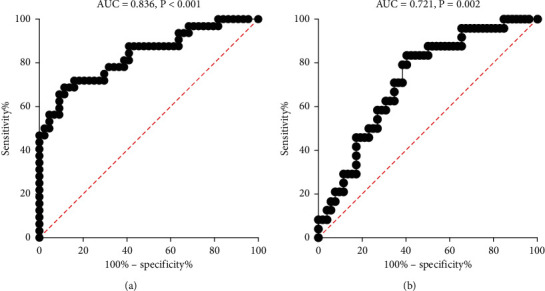
PC ratio ROCs. (a) PC ratio ROC on diagnosing moderate-to-severe pneumonia. (b) PC ratio ROC on diagnosing GI initial GI involvement.

ROC was used to assess the predicting power of PC ratio to assess pulmonary injuries and risk of initial GI involvement (Figure 2). For pulmonary injury, the area under curve is 0.836 (95% CI: 0.742, 0.930, *p* < 0.001) (Figure 2(a)), with a cutoff value at 7.59, while for the risk of initial GI involvement, the area under curve is 0.721 (95% CI: 0.604, 0.839, *p*=0.002) (Figure 2(b)), with a cutoff value at 14.1.

### 3.5. Association between PC Ratio and Other Extrapulmonary Injuries

**Figure 3:**
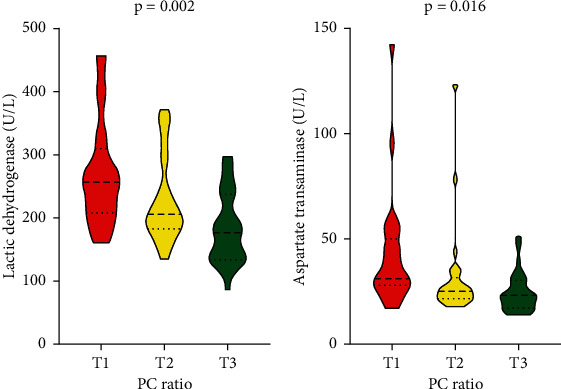
Comparison of multi-organ injuries in PC tertiles.

A violin boxplot was used to illustrate the associations of PC ratio with other extrapulmonary injuries (Figure 3). When compared with middle and top tertile, the bottom tertile of PC ratio showed the highest concentration of lactic dehydrogenase (*p*=0.002) and aspartate transaminase (*p*=0.016), indicating probable occurrence of liver injuries or heart injuries.

## 4. Discussion

Patients infected with SARS-CoV-2 virus exhibit a wide spectrum of clinical manifestations; therefore, the underline mechanisms and our understanding of the clinical and pathologic characteristics are still evolving. Our findings showed for the first time that PC ratio could act as an efficient predictor for COVID-19 associated multi-organ injuries, in the manner of package solution. The PC ratio is inversely associated with the moderate-to-severe chest CT performance, the risk of initial GI involvement, and other extrapulmonary injuries. Notably, no significant difference of age and gender was observed in PC ratio groups (Table 1), which were thought to affect the clinical outcome of COVID-19 patients [11], and proved a safe basis for the present research.

Though several other researches have shown that plasma cytokines released by innate immune cells or adaptive immune cells were positively associated with the progression and severe outcome of COVID-19 patients [12–14], however, there are obviously several obstacles in the application of cytokine family, including being time-consuming, resources-dependent and expensive, especially inconvenient for the patients suffering from poverty. Studies have revealed that circulating platelet exhibits intravascular crawling behavior, promoting formation of thrombus, and bundling pathogen from dissemination in chronic or acute infectious diseases [5, 15].

### 4.1. Correlation between PC Ratio and Pulmonary Manifestations

In this retrospective observational study, a negative correlation between PC ratio and risk of moderate-to-severe pneumonia was found, independent of likely confounders, including age, gender, hypertension, total protein, albumin, lactic dehydrogenase, and lymphocyte (Table 2). Our findings were supported by previous observations. Prior studies showed that COIVD-19 patients showed a higher ratio of immature platelet and circulating platelet decrease when compared with healthy controls [16]. Others found that COVID-19 patients showed a lower platelet reactivity when compared with health control, which might be caused by exhausted mature platelet [16, 17], indicating an inverse correlation between platelet count and function and SARS-CoV-2 virus infection. Our previous studies have shown that plasma CRP level is positively associated with severity of pneumonia in COVID-19 patients [7]. Taken together, this finding in the present study is reasonable.

In order to explore the predicting power of PC ratio on moderate-to-severe pneumonia, ROC curve was applied (Figure 2(a)), which showed a satisfactory power in predicting lung injury.

### 4.2. Correlation between PC Ratio and Risk of Initial GI Involvement

It is well established that the angiotensin-converting enzyme 2 receptor was responsible for SARA-CoV-2 adhesion and invasion, which widely exists in the organs such as pulmonary and GI tract [18, 19]. Our previous study had shown that circulating lymphocyte was inversely associated with the risk of GI involvement [20]. We further explored PC ratio on the risk of GI involvement and found that PC ratio also showed a satisfactory predictive power on the risk of GI involvement (Figure 2(b)).

Logistic regression was further applied to study the correlation between PC ratio and risk of initial GI involvement. When compared to the 1^st^ tertile of PC ratio, the risk of initial GI involvement decreased dramatically in the 3^rd^ tertile (Table 3), which suggested that when PC ratio is over 25.44, the risk of GI involvement decreased dramatically. Our result was further supported by the findings in existing investigations. The natural history of the SARS-CoV-2 is reportedly incubated initially in the pulmonary tract and GI tract, and then recruited, activated platelet, which is congruent to our findings. Interestingly, we did not find a significant difference in positive rate by qRT-PCR in stool between the different tertiles of PC ratio (Table 1), *p*=0.051. This finding should be further validated with larger study population.

### 4.3. Correlation between PC Ratio and Other Extrapulmonary Injuries

As shown in Figure 3, the COVID-19 patients with lower PC ratio showed a significant higher concentration of lactic dehydrogenase (LDH), aspartate transaminase (AST) at baseline, indicating happening of hepatitis, myocardial injury, or multi-organ injuries. Our findings were supported by previous researches. Ze-yang Ding et al. [21] have reported that abnormal AST and direct-bilirubin at admission were positively associated with COVID-19 mortality, indicating AST could be an independent risk factor for disease progression. On the other hand, Liling Zhi et al. [22] have reported that upregulated LDH could act as high-risk factor suggesting myocardial damage in COVID-19. In combination, our finding that PC ratio could predict liver, myocardial injury, or multi-organ injuries in COVID-19.

The major limitation of this study included the relatively small sample size from the two medical centers, which might lead to possible selection bias, while the major strength is that the research is the first study for developing a package solution in predicting COVID-19 severity and multi-organ injuries. In addition, the Asian ethnicity and the lack of medical history in this cohort might influence the generalizability of our results in other ethnic groups. Therefore, the inverse relationship between PC ratio and COVID-19 severities and multi-organ injuries remains to be established in further studies.

## 5. Conclusions

In conclusion, the results of this preliminary study were as follows: (1) PC ratio is inversely correlated with pulmonary injury in CPOVID-19; (2) PC ratio is inversely correlated with GI involvement; (3) PC ratio is inversely correlated with other extrapulmonary organ injuries, especially hepatitis and myocardial injury. Overall, PC ratio could act as the predictor for COVID-19 severity and multi-organ injuries, in the manner of a package solution. The higher the PC ratio, the less severe the COVID-19 patient. We would assess the COVID-19 patients at admission with the PC ratio, for the purpose of screening those with higher risk of severe COVID-19 progression and multi-organ injuries.

## Figures and Tables

**Table 1:** Baseline characteristics of included patients, stratified by PC ratio tertiles.

	T1 (*n* = 25)	T2 (*n* = 25)	T3 (*n* = 26)	*p* value
Demographics				
Age (years)	49.4 ± 11.1	43.3 ± 14.1	41.2 ± 11.9	0.058
Male (%)	14 (56.0)	16 (64.0)	12 (46.2)	0.438
M-S CT performance (%)	21 (84.0)	7 (28.0)	4 (15.4)	<0.001
Highest temperature	38.3 ± 0.6	38.2 ± 0.4	37.9 ± 0.8	0.039
Pulse per minute	93 ± 12	92 ± 11	91 ± 10	0.763
Hypertension (%)	10 (40.0)	4 (16.0)	4 (15.4)	0.064
Fatigue (%)	15 (60.0)	11 (44.0)	11 (42.3)	0.382
Type II diabetes (%)	2 (8.0)	1 (4.0)	1 (3.8)	NA
Hypertension (%)	10 (40.0)	4 (16.0)	4 (15.4)	0.064
GI manifestation				
Vomiting (%)	7 (28.0)	4 (16.0)	2 (7.7)	0.154
Diarrhea (%)	8 (32.0)	7 (28.0)	2 (7.7)	0.081
Initial GI involvement (%)	12 (48.0)	9 (36.0)	3 (11.5)	0.017
Respiratory manifestation				
Breath (times per minute) (%)				0.466
16	0 (0.0)	0 (0.0)	1 (3.8)	
17	0 (0.00)	1 (4.0)	0 (0.0)	
18	1 (4.0)	1 (4.0)	3 (11.5)	
19	2 (8.0)	3 (12.0)	0 (0.0)	
20	16 (64.0)	17 (68.0)	20 (76.9)	
21	1 (4.0)	1 (4.0)	1 (3.8)	
22	4 (16.0)	2 (8.0)	0 (0.0)	
23	1 (4.0)	0 (0.0)	1 (3.8)	
Dyspnea (%)	9 (36.0)	4 (16.0)	1 (3.8)	0.012
Cough (%)	17 (68.0)	17 (68.0)	16 (61.5)	0.853
Laboratory characteristics				
WBC count (10^9/l)	4.21 ± 1.20	4.60 ± 1.69	4.54 ± 1.56	0.605
Neutrophil count (10^9/l)	2.99 ± 0.85	2.98 ± 1.41	2.82 ± 1.35	0.857
Lymphocyte count (10^9/l)	0.89 ± 0.37	1.21 ± 0.47	1.27 ± 0.60	0.017
Hemoglobulin (g/l)	133.64 ± 14.44	140.00 ± 12.78	135.81 ± 11.25	0.213
Total protein (g/l)	71.00 ± 6.71	69.64 ± 4.62	72.22 ± 5.68	0.284
Globulin (g/l)	31.45 ± 5.30	28.04 ± 3.65	29.69 ± 5.19	0.046
Albumin (g/l)	39.54 ± 3.63	41.60 ± 3.72	42.53 ± 2.27	0.005
Lactic dehydrogenase (U/L)	270.08 ± 83.58	227.36 ± 69.01	183.46 ± 56.63	<0.001
Creatinine (mmol/l)	72.24 ± 19.16	73.73 ± 16.56	66.92 ± 12.81	0.299
Alanine aminotransferase (U/L)	37.24 ± 23.94	29.64 ± 30.62	23.62 ± 20.17	0.162
Aspartate transaminase (U/L)	41.84 ± 26.89	31.64 ± 22.55	24.65 ± 9.60	0.016
Total bilirubin (mmol/l)	15.20 ± 9.43	14.48 ± 9.39	14.84 ± 6.53	0.957
qRT-PCR on stool (%)	6 (24.0)	9 (36.0)	2 (7.7)	0.051

*Note.* qRT-PCR: quantitative real-time polymerase chain reaction; GI: gastrointestinal; PC ratio: platelet-to-C-reactive protein ratio; PC ratio tertile 1 ≤ 7.60; 7.60 PC ratio tertile 2 ≤ 25.44; PC ratio tertile 3 > 25.44. M-S CT performance: moderate-to-severe computed tomography performance. NA: not applicable. Data are presented as means ± SD, or *n* (%).

**Table 2:** Relationship between PC ratio and chest CT performance.

PC ratio tertiles	Chest CT performance					
	Crude model		Adjusted model I		Adjusted model II	
	OR (95% CI)	*p* value	OR (95% CI)	*p* value	OR (95% CI)	*p* value
1	0		0		0	
2	−0.76 (−1.03, −0.49)	<0.001	−0.69 (−0.96, −0.43)	<0.001	−0.51 (−0.78, −0.24)	<0.001
3	−0.89 (−1.15, −0.62)	<0.001	−0.80 (−1.07, −0.53)	<0.001	−0.53 (−0.82, −0.24)	<0.001

Crude model adjusted for none. Adjusted model I adjusted for age, gender. Adjusted model II adjusted for age, gender, hypertension, total protein, albumin, lymphocyte, lactic dehydrogenase, lymphocyte. PC ratio tertile 1 ≤ 7.60; 7.60 PC ratio tertile 2 ≤ 25.44; PC ratio tertile 3 > 25.44.

**Table 3:** Relationship between PC ratio and initial GI involvement.

PC ratio tertiles	Initial GI involvement					
	Crude model		Adjusted model I		Adjusted model II	
	OR (95% CI)	*p* value	OR (95% CI)	*p* value	OR (95% CI)	*p* value
1	1		1		1	
2	0.61 (0.20, 1.89)	0.391	0.57 (0.18, 1.83)	0.345	0.94 (0.24, 3.59)	0.923
3	0.14 (0.03, 0.59)	0.008	0.13 (0.03, 0.58)	0.007	0.18 (0.03, 0.97)	0.005

Crude model adjusted for none. Adjusted model I adjusted for age, gender. Adjusted model II adjusted for age, gender, hypertension, total protein, albumin, lymphocyte, lactic dehydrogenase, lymphocyte. PC ratio tertile 1 ≤ 7.60; 7.60 < PC ratio tertile 2 ≤ 25.44; PC ratio tertile 3 > 25.44.

## Data Availability

Based on reasonable request, the datasets could be achieved after published online.

## Abbreviations

SARS-CoV-2: Severe acute respiratory syndrome coronavirus 2
COVID-19: Coronavirus 2019
CI: Confidence interval
RT-PCR: Reverse transcriptase-polymerase chain reaction
CDCP: Center for Disease Control and Prevention
GI: Gastrointestinal
CT: Computed tomography
PC ratio: Platelet-to-C-reactive protein ratio
CT: Computed tomography.
